# CRUNC: a cryopreservation method for unencapsulated gemmae of *Marchantia polymorpha*

**DOI:** 10.7717/peerj.10174

**Published:** 2020-10-23

**Authors:** Hitomi Takahashi, Yutaka Kodama

**Affiliations:** Center for Bioscience Research and Education, Utsunomiya University, Utsunomiya, Japan

**Keywords:** Bryophyte, Cryoconservation, Liverworts, Unencapsulation, Gemma

## Abstract

Genetic modifications such as mutation and transformation are powerful tools to study the function of genes and proteins in the model liverwort *Marchantia polymorpha*, but maintaining the resulting germplasm requires a practical, reliable method. Cryopreservation methods allow researchers to maintain mutant and transgenic lines of *M. polymorpha*. To date, two methods have been developed for cryopreservation of *M. polymorpha* gemmae: in the first method, unencapsulated gemmae are stored in liquid nitrogen at −­196 °C, and in the second method, encapsulated gemmae are stored in liquid nitrogen at −­196 °C or a deep freezer at −80 °C. In the present study, we developed a simple method named CRUNC (cr yopreservation of un en c apsulated gemmae), which can be used to store unencapsulated, dried gemmae of wild-type and transgenic *M. polymorpha* lines in liquid nitrogen and in freezers at −80 °C and −20 °C. Using the CRUNC method, we observed a high recovery rate (as high as 100%) and successful long-term (5 months) storage of the gemmae. Therefore, the CRUNC method is practical for maintaining valuable *M. polymorpha* germplasm.

## Introduction

Genetic transformation, gene targeting, and genome editing methods have been developed in the model liverwort *Marchantia polymorpha* to analyze gene/protein function (Nasu et al., 1997; Chiyoda et al., 2007; Ishizaki et al., 2008, 2013; Kubota et al., 2013; Sugano et al., 2014, 2018; Tsuboyama & Kodama, 2014, 2018a, 2018b; Tsuboyama-Tanaka & Kodama, 2015; Tsuboyama-Tanaka, Nonaka & Kodama, 2015; Tsuboyama et al., 2018). As research progresses, the number of valuable wild-type (WT), mutant, and transgenic lines of *M. polymorpha* that need to be maintained increases. Because *M. polymorpha* is a dioecious liverwort, the first filial generation spores from crosses of male and female strains contain diverse genetic information, making it impossible to maintain spores of *M. polymorpha* with the same genetic information as their parents. Therefore, maintaining valuable *M. polymorpha* germplasm requires an easy and efficient storage method.

Three methods for cryopreservation of *M. polymorpha* (two for gemmae and one for spermatozoa) have been reported: cryopreservation of unencapsulated gemmae (Wu et al., 2015), encapsulated gemmae (Tanaka et al., 2016), and spermatozoa (Togawa et al., 2018). The unencapsulated gemmae are placed on a small filter paper envelope, dried in a box filled with silica gel, and then cryopreserved in liquid nitrogen at −196 °C (Wu et al., 2015). This method does not require encapsulation of gemmae, but it requires filter paper envelopes, large amounts of silica gel, and liquid nitrogen (Wu et al., 2015). For encapsulation methods, gemmae are encapsulated with calcium alginate gel, and then dehydrated (Tanaka et al., 2016). The dried, encapsulated gemmae can then be cryopreserved in liquid nitrogen or in a deep freezer at −80 °C (Tanaka et al., 2016). For spermatozoa of male *M. polymorpha*, the spermatozoa are suspended in a cryoprotective solution in the appropriate container for freezing cells, frozen in a deep freezer, and then cryopreserved in liquid nitrogen (Togawa et al., 2018). Among these three methods, cryopreservation of unencapsulated gemmae (Wu et al., 2015) may require the least amount of preparation. Notably, the above three methods were tested only using WT strains, but not mutant and transgenic lines. Although each method has advantages and disadvantages, developing multiple storage methods is important for effectively storing *M. polymorpha* lines.

In the present study, we developed a simple storage method, named CRUNC (cryopreservation of unencapsulated gemmae), to store dried, unencapsulated gemmae of WT and transgenic lines in liquid nitrogen at −196 °C, in a deep freezer at –80 °C, or in a freezer at –20 °C. We show here that the CRUNC method is practical for storing valuable lines of *M. polymorpha*.

## Materials and Methods

### Plant materials and culture condition

Thalli of a WT strain (female BC3-38 strain) and four transgenic lines (TG#060-1, TG#066-5, TG#164-3, and TG#253-6) of *Marchantia polymorpha* were asexually cultured on half-strength B5 (1/2 B5) agar medium under 75 µmol photons m^−2^ s^−1^ continuous white light (FL40SW; NEC Corporation, Tokyo, Japan) in a culture room at 22 °C. The white light intensity was measured using an LI-250A light meter (LI-COR Biosciences, Lincoln, NE, USA). These four transgenic lines were previously reported (Table 1) (Kimura & Kodama, 2016; Sakata et al., 2019; Fujii et al., 2020). The transgenic lines were maintained at the G1 generation in the culture room, and G2 gemmae were used in the following experiments.

**Table 1:** Transgenic lines tested in this study.

Name	Binary vector	Genetic background	References
TG#060-1	pMpGWB105-MpTublin	Tak-1 (WT)	Kimura & Kodama (2016)
TG#066-5	pMpGWB403-Lifeact-Citrine	Tak-1 (WT)	Kimura & Kodama (2016)
TG#164-3	pMpGWB306-MpPHOT^D922N^	Mp*phot*^KO^	Sakata et al. (2019)
TG#253-6	pMpGWB102-Citrine	Tak-1 (WT)	Fujii et al. (2020)

### Sterilization of silica-gel beads

Silica-gel beads (6 mesh up, No. 19000535; Hayashi Pure Chemical Ind., Ltd., Osaka, Japan) in a glass Petri dish (MINIP-3; Sansyo Co., Ltd., Tokyo, Japan) were sterilized by dry heat in an oven (WFO-400; EYELA, Tokyo, Japan) at 180 °C for 1 h, and then placed in a laminar flow hood (VCB-1300; Oriental Giken Inc., Tokyo, Japan) until use.

### Calculation of recovery rate and dehydration rate

The recovery rate (%R) was calculated by dividing the number of gemmae recovered (NR) by the number of all gemmae tested (NT): %R = (NR/NT) × 100. The dehydration rate (%D) was obtained by dividing the weight of gemmae after dehydration (WA) by the weight of gemmae before dehydration (WB): %D = (1-(WA/WB)) × 100. For quantitative analysis, average and standard deviation were calculated from three experiments (see raw data in Supplemental File 1).

## Results and discussion

### Basic CRUNC procedure

**Figure 1:**
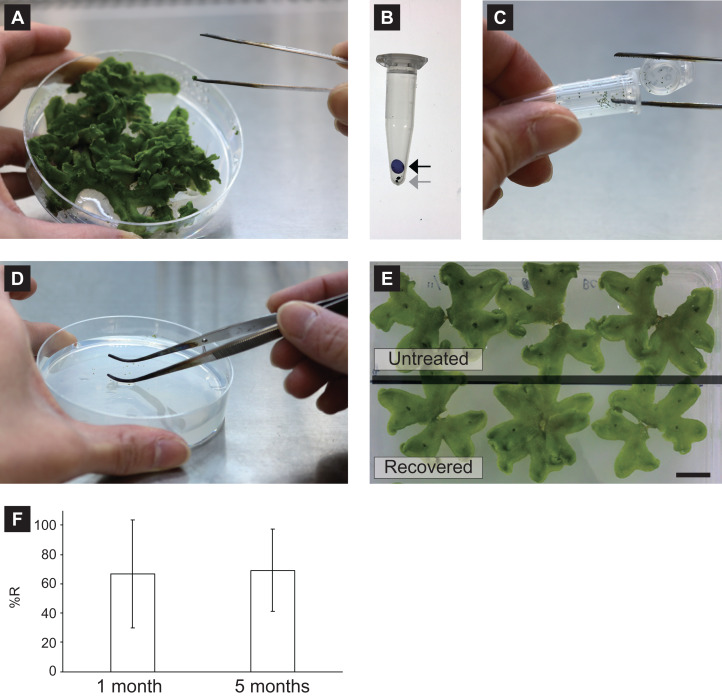
Snapshots of the CRUNC procedure. (A) Collection of gemmae using straight tweezers. (B) Putting a silica-gel bead into the 1.5-mL tube containing gemmae. Black and gray arrows indicate a silica-gel bead and gemmae, respectively. (C) Picking gemmae from the 1.5-mL tube. (D) Thawing frozen gemmae with sterile ultra-pure water on agar medium for recovery. (E) Growth comparison between recovered gemmae and untreated gemmae. Gemmae were cultured on half-strength B5 agar medium in a rectangular Petri dish for 18 days. (F) The recovery rate (%R) of unencapsulated gemmae cryopreserved for 1 month and 5 months. Gemmae from the same gemma cup were divided into two groups and cryopreserved at −80 °C for 1 month and 5 months, and the recovery rates were evaluated.

All equipment, products, and reagents used in this study are listed in Tables 2 and 3. To cryopreserve gemmae by the CRUNC method, we collected the gemmae from two gemma cups (approximately 100–300 gemmae) using straight tweezers under a laminar flow hood (Fig. 1A). The gemmae were put into a 1.5-mL tube under the hood, and the weight of the gemmae was measured with an electric balance. To dry the gemmae, a bead of silica gel was added to the tube using straight tweezers under the hood (Fig. 1B), and the capped tube was incubated on the benchtop at 22 °C for 1 h. To evaluate the degree of drying, the silica-gel bead was removed as shown in Video 1 and the weight of the gemmae in the tube was measured again.

**Table 2:** Equipment list.

Equipment	Use
Laminar flow hood	General manipulations for the CRUNC method
Micropipette and Micropipette tips (in a laminar flow hood)	Dropping sterile ultrapure water onto the solid medium when gemmae were thawed at the recovery step
Gas burner (in a laminar flow hood)	Sterilization of tweezers
Straight tweezers (in a laminar flow hood)	Putting gemmae or a silica-gel bead into a 1.5-mL tube, and picking them out from the 1.5-mL tube
Curved tweezers (in the laminar flow hood)	Streaking gemmae when gemmae were thawed at the recovery step
Oven	Sterilization of silica-gel beads
pH meter	Making solid medium
Electric balance	Weighing gemmae in tube
Non-frost-free freezer (−20 °C)	Cryopreservation at −20 °C
Deep freezer (−80 °C)	Cryopreservation at −80 °C
Incubator (28 °C)	Thawing step in the freeze-thaw-freeze treatment

**Table 3:** Products and reagents list.

Product name	Manufacturer	Product ID number
1.5-mL tubes	WATSON	131-7155C
Silica gel	Hayashi Pure Chemical	19000535
Glass Petri dish	SANSYO	MINIP-3
Gamborg’s B5 medium salt mixture	Wako	399-00621
KOH	Wako	168-03855
MES	Nacalai tesque	21623-26
Agar	SSK sales	BOP
ø90 × 20 mm Petri dish	AS ONE	1-8549-04
Rectangular Petri dish	Eiken Chemical	AW2000
Micropore tape (25-mm wide)	3M	1530-1

After the gemmae were subjected to the drying process, the tube containing the gemmae without the silica-gel bead was directly transferred into the freezer at −80 °C for cryopreservation. To recover the cryopreserved gemmae, the gemmae were removed from the tube using straight tweezers under the hood (Fig. 1C), thawed with 200 µL sterile ultra-pure water, spread with curved tweezers on 1/2 B5 agar medium (Fig. 1D; Video 2), and then cultured in the culture room with continuous white light (75 µmol photons m^−2^ s^−1^) at 22 °C. Growth of the recovered gemmae was comparable to that of untreated gemmae (Fig. 1E).

The CRUNC method can be used to store unencapsulated gemmae for a long time. For example, when gemmae from the same gemma cup were divided into two groups and cryopreserved at −80 °C for 1 month and 5 months, the recovery rate (abbreviated %R) was unchanged between the two storage times (Fig. 1F).

### A higher dehydration rate gives a higher efficiency of recovery

**Figure 2:**
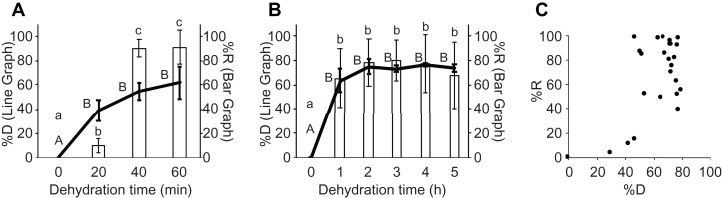
Comparison between the dehydration rate and recovery rate. (A) Comparison between the dehydration rate and recovery rate after dehydration with a silica-gel bead for 60 min. Left (line graph) and right (bar graph) Y-axes show the dehydration rate (%D) and recovery rate (%R), respectively. Different uppercase (A and B for line graph) and lowercase letters (a, b, c for bar graph) indicate a significant difference (Tukey’s test; *p* < 0.05). (B) Comparison between the dehydration rate and recovery rate after dehydration with a silica-gel bead for 5 h. Left (line graph) and right (bar graph) *Y*-axes show the dehydration rate (%D) and recovery rate (%R), respectively. Different uppercase (A and B for line graph) and lowercase letters (a, b for bar graph) indicate a significant difference (Tukey’s test; *p* < 0.05). (C) A scatter plot to compare the dehydration rate (%D) and recovery rate (%R). The recovery rates of 30 experiments are plotted in the graph.

To determine the appropriate degree of drying of the gemmae for maximum recovery and survival, we compared the recovery rates of the gemmae at various levels of dehydration (abbreviated as %D). After incubation with the silica gel for 0, 20, 40, and 60 min, average dehydration rates were 0, 39.2, 54.8, and 62.0%D, respectively (Fig. 2A). After −80 °C cryopreservation of the dehydrated gemmae for 1 day, the average recovery rates were 0, 10.1, 90.4, and 91.1%R, respectively, indicating that a higher dehydration rate confers a higher recovery rate (Fig. 2A). When the gemmae were incubated with the silica gel for 5 h, both dehydration and recovery rates remained high (Fig. 2B). A scatter plot of the average recovery rates (30 experiments) shows that 50–80%D was required to give recovery rates above 80%R (Fig. 2C).

In a previous study that explored cryopreservation of unencapsulated gemmae, only dehydration time, but not dehydration rate, was evaluated (Wu et al., 2015). When Wu et al. (2015) dehydrated gemmae for 0, 3, 5, and 7 h, the recovery rates after cryopreservation for 1 day at −196 °C were 0, 68.4, 60.1, and 29.2%R, respectively. In their method, dehydration for 7 h significantly decreased the recovery rate (i.e., 29.2%R). In addition, a combination of dehydration for 5 h with cryopreservation for 2.5 months (75 days) also gave low recovery rates (18.9%R). In the CRUNC method, we focused not only on the dehydration time but also on the dehydration rate, and found that a higher dehydration rate gives a higher recovery rate (Figs. 2A–2C). When we tested dehydration of the gemmae for 24 h in the CRUNC method, the recovery rate was not decreased compared with shorter dehydration times (Fig. S1). Although the dehydration rates of the gemmae in the previous study are unknown (Wu et al., 2015), the recovery rate of the gemmae in the CRUNC method was higher than that of the previously described method. By contrast, a method for cryopreservation of encapsulated gemmae stably achieved 100% (Tanaka et al., 2016), while the CRUNC method achieved 100%R in only some tests (see raw data in Supplemental File 1). Therefore, the CRUNC method requires further improvement.

### Gemmae from younger and older gemma cups are not suitable for the CRUNC method

**Figure 3:**
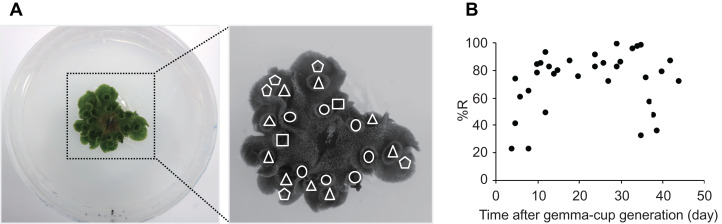
The recovery rates of gemmae from different aged gemma cups. (A) Representative images of thalli with different aged gemma cups. Left image: the thalli were cultured in a 90-mm Petri dish. Right image: enlarged image is shown. Different shapes indicate the positions of different aged gemma cups: circle, triangle, square, and pentagon indicate 9, 7, 5, and 1 day(s) after the generation of the gemma cup, respectively. (B) A scatter plot that shows the recovery rates (%R) of gemmae from different aged gemma cups. The recovery rates of 34 experiments are plotted in the graph.

Thalli of *M. polymorpha* grow radially with repeated dichotomous branching at the apex of each thallus, and a single gemma cup is generated on each branch of the thallus (Shimamura, 2016). Therefore, there are different aged gemma cups on multiple thalli of an individual *M. polymorpha* plant. To determine which gemma cups are most appropriate for the CRUNC method, we put a single gemma on agar medium, observed the growth and generation of gemma cups for 2 months, and then collected gemmae from the different aged gemma cups at the same time (Fig. 3A). The recovery rates of gemmae from younger (under 8 days after generation) and older (over 35 days after generation) gemma cups varied (Fig. 3B). The recovery rate was highest with gemmae from gemma cups at 10–33 days after generation (Fig. 3B). Therefore, gemmae taken from gemma cups at 10–33 days after generation should be used for the CRUNC method.

### Dehydrated, unencapsulated gemmae can be cryopreserved in liquid nitrogen and frozen at −80 °C and −20 °C

**Figure 4:**
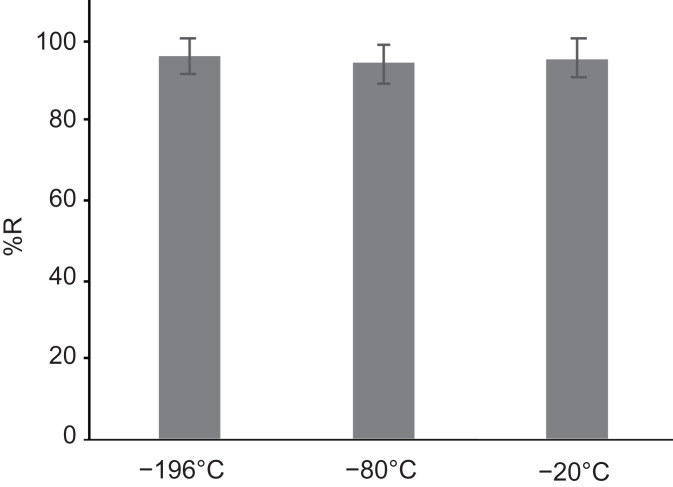
The recovery rates of gemmae cryopreserved in different freezing conditions. Liquid nitrogen (−196 °C), deep freezer (−80 °C), and conventional freezer (−20 °C). There was no significant difference (Tukey’s test; *p* > 0.05).

Previous studies employed liquid nitrogen at −196 °C and a deep freezer at −80 °C to cryopreserve dehydrated unencapsulated and encapsulated gemmae (Wu et al., 2015; Tanaka et al., 2016). For the CRUNC method, we compared liquid nitrogen and freezer storage (deep freezer at −80 °C as well as a non-frost-free freezer at −20 °C). After dehydration of gemmae with a silica-gel bead, the unencapsulated gemmae were directly transferred to the three storage conditions and incubated for 1 day. There was no significant difference among the recovery rates in the three cryopreservation conditions (Fig. 4). These results show that dehydrated gemmae can be cryopreserved in liquid nitrogen (−196 °C), a deep freezer (−80 °C), or a freezer (−20 °C).

### Gemmae cryopreserved by the CRUNC method can tolerate a freeze-thaw-freeze treatment

**Figure 5:**
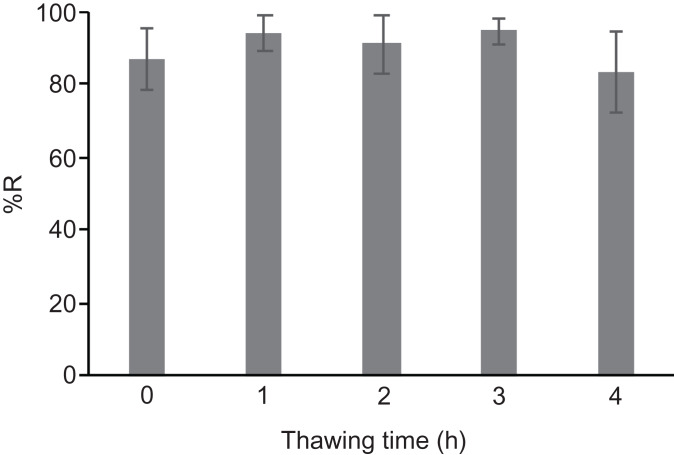
Effect of the freeze-thaw-freeze treatment. The gemmae cryopreserved at −80 °C were then thawed at 28 °C for the indicated times, followed by re-freezing at −80 °C. There was no significant difference (Tukey’s test; *p* > 0.05).

Freeze-thaw cycles can occur unexpectedly during a power outage. To determine the effect of thawing and refreezing of cryopreserved gemmae, we conducted a freeze-thaw-freeze treatment. After cryopreservation of the gemmae, the tube was thawed in an incubator at 28 °C for 1, 2, 3, and 4 h, followed by re-freezing at −80 °C. The recovery rate was unchanged by the thawing and refreezing treatment (Fig. 5), indicating that gemmae cryopreserved by the CRUNC method can survive a short-term thaw and refreezing. Note that this experiment mimics a power outage in non-frost-free freezers; because frost-free freezers undergo frequent freeze-thaw cycles, we recommend the use of non-frost-free freezers for the CRUNC method.

### The recovery rate depends on the transgenic line

**Figure 6:**
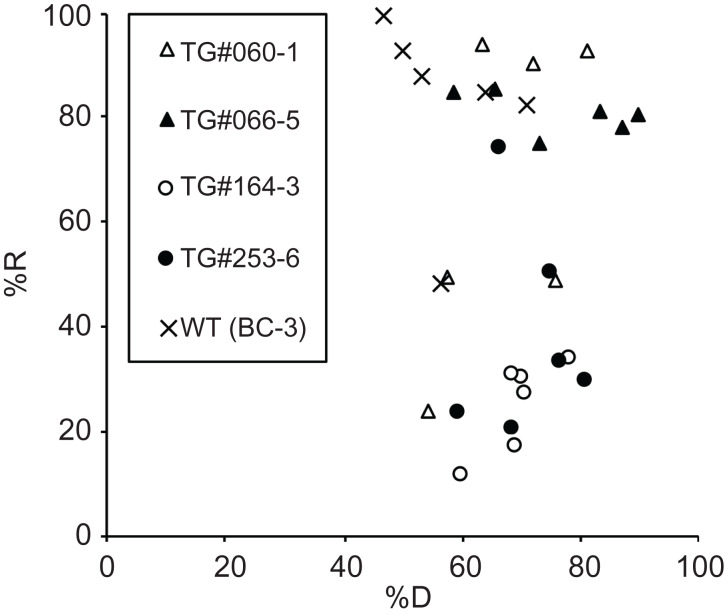
The recovery rates of gemmae of transgenic *M. polymorpha*. A scatter plot that shows the dehydration rate (%D) and recovery rate (%R) of the four different transgenic lines (TG#060-1, TG#066-5, TG#164-3, TG#253-6). The WT line (BC3-38) was used as a control. The recovery rates of six experiments for each line (a total of 30 experiments) are plotted in the graph.

In the above experiments, WT gemmae of *M. polymorpha* (the BC3-38 strain) were used to develop the CRUNC method. Because the purpose of the present study is to improve the cryopreservation of genetically modified lines, we tested the CRUNC method on gemmae from four different transgenic lines (TG#060-1, TG#066-5, TG#164-3, and TG#253-6) that we reported previously (Table 1). We conducted six replicate experiments for each line and found that compared to the BC3-38 strain, the recovery rate of the transgenic gemmae varied, and was generally lower than that of the WT (Fig. 6). The varied recovery rate of transgenic gemmae suggested that the function(s) of the transgene(s) and/or the integration position of transfer-DNA in the genome might affect the fitness of the individual lines and thus their recovery rate. Therefore, for effective long-term storage of transgenic lines, a pre-check of the recovery rate is recommended.

Although the molecular mechanisms underlying the successful recovery of dehydrated and unencapsulated gemmae after cryopreservation remain to be determined, *M. polymorpha* does have natural dehydration and freezing resistance to survive dry and cold conditions during winter. Therefore, combining *Agrobacterium*-mediated genetic transformation with the CRUNC method might allow us to screen for genes involved in freezing resistance and recovery. These genes could be used to improve the survival of gemmae after dehydration and cryopreservation.

## Conclusions

We report the development of the CRUNC method for simple and effective cryopreservation of unencapsulated gemmae of *M. polymorpha*. Compared with the cryopreservation method of unencapsulated gemmae (Wu et al., 2015), the CRUNC method does not require the filter paper envelope, large amounts of silica gel, and liquid nitrogen. Using the CRUNC method, we successfully cryopreserved a WT line and four transgenic lines. Because the three previous methods to cryopreserve *M. polymorpha* (two for gemmae and one for spermatozoa) were tested only using WT strains (Wu et al., 2015; Tanaka et al., 2016; Togawa et al., 2018), the present study is the first report on cryopreservation of transgenic lines. In the CRUNC method, the recovery rate was very high (as high as 100%), even after a storage period of 5 months. To fully prove its utility, the CRUNC method will need to be tested for storage periods longer than 5 months. However, given that the recovery rate was unchanged even after 5 months of storage, we believe that the CRUNC method can be used for much longer storage periods. Furthermore, in the CRUNC method, three types of storage conditions (i.e., liquid nitrogen at −196 °C, a deep freezer at −80 °C, and a non-frost-free freezer at −20 °C) can be used, and the gemmae cryopreserved by the CRUNC method tolerate a short-term freeze-thaw-freeze treatment. Therefore, the CRUNC method is practical for maintaining various valuable lines of *M. polymorpha*.

## Supplemental Information

10.7717/peerj.10174/supp-1 Supplemental Information 1 Raw Data.

10.7717/peerj.10174/supp-2 Supplemental Information 2 The recovery rates of gemmae in the CRUNC method with dehydration for 24 h. A scatter plot to compare between the dehydration rate (%D) and recovery rate (%R).

10.7717/peerj.10174/supp-3 Supplemental Information 3 Removing a silica-gel bead from the 1.5-mL tube containing gemmae before measurement of the weight of the gemmae.

10.7717/peerj.10174/supp-4 Supplemental Information 4 Thawing frozen gemmae using sterile ultra-pure water on agar medium for recovery.

## Additional Information and Declarations

### Data Availability

The following information was supplied regarding data availability: The raw measurements are available in the Supplemental File.
